# Risk factors for pulmonary cavitation in tuberculosis patients from China

**DOI:** 10.1038/emi.2016.111

**Published:** 2016-10-12

**Authors:** Liqun Zhang, Yu Pang, Xia Yu, Yufeng Wang, Jie Lu, Mengqiu Gao, Hairong Huang, Yanlin Zhao

**Affiliations:** 1 Beijing Chest Hospital, Capital Medical University, Beijing Tuberculosis and Thoracic Tumor Research Institute, Beijing 101149, China; 2 National Center for Tuberculosis Control and Prevention, Chinese Center for Disease Control and Prevention, Beijing 102206, China; 3 Beijing Key Laboratory for Pediatric Otolaryngology, Head and Neck Science, Beijing Pediatric Research Institute, Beijing Children's Hospital, Capital Medical University, Beijing 100045, China

**Keywords:** cavitary, molecular characteristics, *Mycobacterium tuberculosis*, risk factor

## Abstract

Pulmonary cavitation is one of the most frequently observed clinical characteristics in tuberculosis (TB). The objective of this study was to investigate the potential risk factors associated with cavitary TB in China. A total of 385 smear-positive patients were enrolled in the study, including 192 (49.9%) patients with cavitation as determined by radiographic findings. Statistical analysis revealed that the distribution of patients with diabetes in the cavitary group was significantly higher than that in the non-cavitary group (adjusted odds ratio (OR) (95% confidence interval (CI)):12.08 (5.75–25.35), *P*<0.001). Similarly, we also found that the proportion of individuals with multidrug-resistant TB in the cavitary group was also higher than that in the non-cavitary group (adjusted OR (95% CI): 2.48 (1.52–4.07), *P*<0.001). Of the 385 *Mycobacterium tuberculosis* strains, 330 strains (85.7%) were classified as the Beijing genotype, which included 260 strains that belonged to the modern Beijing sublineage and 70 to the ancient Beijing sublineage. In addition, there were 80 and 31 strains belonging to large and small clusters, respectively. Statistical analysis revealed that cavitary disease was observed more frequently among the large clusters than the small clusters (*P*=0.037). In conclusion, our findings demonstrate that diabetes and multidrug resistance are risk factors associated with cavitary TB. In addition, there was no significant difference in the cavitary presentation between patients infected with the Beijing genotype strains and those infected with the non-Beijing genotype strains.

## Introduction

Tuberculosis (TB), which is caused by the *Mycobacterium tuberculosis* (MTB) complex, remains a major public health threat worldwide.^1, 2^ According to the World Health Organization, there were 9.6 million new TB cases and 1.2 million deaths from TB in 2014.^1^ China has the world's second largest population of TB patients (after India), with >1 million new cases of TB each year.^1^ The fifth national TB prevalence survey conducted in 2010 indicates that China has an estimated prevalence of 442 cases per 100 000 population.^2^ Although the prevalence of TB has decreased by more than half from 1990 to 2010, TB is still a major public health and socio-economic issue in China.^3, 4^

Pulmonary cavitation is one of the most frequently observed clinical characteristics in TB, accounting for >40% of adults with pulmonary TB at the time of diagnosis.^5, 6, 7^ Canetti and Grosset compared the bacillary load in lung sections from resected lung tissues of pulmonary TB patients, and their findings revealed that the bacillary load in the cavity walls was 10^5^ times higher than that in the caseous necrosis.^8^ Several laboratory examinations have also demonstrated that cavitation is associated with higher bacterial load in the sputum, which may serve as a strong indicator for early treatment.^9, 10, 11^ The presence of cavitary disease is associated with treatment failure and relapse among pulmonary TB patients.^12, 13^

Variable number of tandem repeats (VNTR) genotyping is the international gold standard for typing of mycobacterial isolates, including MTB and several non-tuberculous mycobacteria species.^14, 15^ A study investigating the relationship between VNTR profiles and disease progression revealed that specific VNTR genotypes were associated with disease progression in *Mycobacetrium avium* pulmonary infection.^16^ Similar results were observed by Shin *et al.*^17^ who showed that fibrocavitary diseases were more likely to be grouped into a particular cluster among *Mycobacterium abscessus* diseases. However, few studies have examined the potential association between the MTB genotype and the cavitary phenotype. In this study, we aimed to determine the potential risk factors associated with cavitary TB and investigated whether there was relationship between the MTB genotype and cavitary disease in China.

## Materials and Methods

### Ethics statement

This study was approved by the Ethics Committee of the Beijing Chest Hospital, which is affiliated with Capital Medical University. The patients signed informed consent forms before they were enrolled in the study.

### Patient enrollment

Between November 2013 and December 2014, a total of 467 smear-positive pulmonary TB patients sought health care in the Beijing Chest Hospital. Patients infected with non-tuberculous mycobacteria (*n*=32), patients with contaminated culture results (*n*=21) and patients who refused to participate this study (*n*=29) were excluded. Finally, 385 patients were enrolled in this study. Demographic information was obtained from the informed consent forms. The chest radiography and high-resolution computed tomography findings were classified at the time of diagnosis. All patients were negative for HIV. The sputum samples collected from the patients were transferred to the clinical laboratory at Beijing Chest Hospital for culturing. Bacterial cells were isolated from Löwenstein–Jensen medium.

### Genomic DNA extraction

Genomic DNA was purified from freshly cultured mycobacteria using previously described methods.^18^ Briefly, the fresh bacteria cells were harvested from Löwenstein–Jensen medium and then transferred to a microcentrifuge tube with 500 μL of Tris-EDTA (TE) buffer. After centrifugation at 13 000 r.p.m. for 2 min, the supernatant was discarded and the pellet was resuspended in 500 μL TE buffer. The suspension was incubated in a 95 °C water bath for 1 h and then centrifuged at 13 000 r.p.m. for 5 min to remove the cellular debris. The crude DNA in the supernatant was used as the PCR template.

### Drug susceptibility testing

The absolute concentration method was used to perform drug susceptibility testing for isoniazid, rifampicin, ethambutol, streptomycin, ofloxacin and kanamycin as previously reported,^19^ and the concentration of the drugs in the media were as follows: rifampicin, 40 μg/mL; isoniazid, 10 μg/mL; ethambutol, 0.2 μg/mL; streptomycin, 2 μg/mL; ofloxacin, 2 μg/mL; and kanamycin, 30 μg/mL. Multidrug-resistant TB (MDR-TB) strains were defined as those with resistance to both isoniazid and rifampicin.

### Genotyping

The identification of genomic deletions in the RD105 region was performed using real-time PCR to distinguish the Beijing genotype from the non-Beijing genotype as previously reported.^20^ The strains with amplification of the RD105 region were classified into the non-Beijing genotype, whereas the strains with no amplification of the RD105 region belonged to the Beijing genotype. The presence of the IS6110 insertion in the NTF region was analyzed to distinguish the modern Beijing strains from the ancient Beijing strains according to previously described methods.^21^

In addition, the classical 15-locus VNTR typing method was performed to determine the molecular characteristics of the strains isolated from the patients according to previously described methods.^22^ The PCR mixture was prepared in a volume of 20 μL containing 10 μL of 2 × PCR Mix (Genestar, Beijing, China), 2 μL of crude DNA template and 0.2 μM of each primer. PCR was carried out under the following conditions: one cycle at 94 °C for 5 min for initial denaturation, and then 35 cycles at 94 °C for 1 min, 60 °C for 1 min and 72 °C for 1 min, followed by one cycle at 72 °C for 10 min for a final extension. The size of the amplicons was determined using agarose electrophoresis. In addition, the discriminatory power of each mycobacterial interspersed repetitive unit (MIRU)-VNTR locus was evaluated using the Hunter–Gaston Discriminatory Index (HGDI). The HGDI and clustering rate were analyzed as previously described.^18, 23^ A large cluster was defined as a cluster with more than five strains, and a small cluster was defined as a cluster with fewer than five strains.

### Data analysis

We used the BioNumerics software version 5.0 (Applied Maths, Sint-Martens-Latem, Belgium) to perform the minimum spanning tree analysis. The chi-square or Fisher's exact test was used to evaluate the associations among the categorical variables. In addition, the odds ratio (OR) and 95% confidence interval (CI) were utilized to express the statistical results. The multivariate models were obtained using forward stepwise logistic regression procedures to determine whether covariates that were statistically significant in the univariate analysis were independently associated with cavitary TB. All calculations in this study were carried out using the SPSS program (SPSS 11.5 version for Windows, SPSS Inc., Chicago, IL, USA). *P*<0.05 was considered statistically significant.

## Results

### Socio-demographic and clinical characteristics of patients

A total of 385 TB smear-positive patients were enrolled in this study, including 192 (49.9%) patients with cavitation, as determined by radiographic findings. We first compared the socio-demographic characteristics between patients with cavitary disease and non-cavitary disease. As shown in Table 1, the percentage of patients aged 45–64 years was significantly higher in the cavitary TB group than in the non-cavitary TB group (OR (95% CI): 3.03 (1.60–5.74), *P*=0.001), whereas there were no significant differences in the other age groups between the groups. In addition, the statistical analysis revealed no statistically significant differences in the other socio-demographic characteristics, including gender and treatment history.

We further compared the comorbidities and drug susceptibility profiles of cavitary and non-cavitary patients to determine the potential risk factors for cavitary TB infection. Interestingly, the distribution of patients with diabetes in the cavitary TB group was significantly higher than that in the non-cavitary TB group (OR (95% CI): 12.25 (5.89–25.48), *P*<0.001). Similarly, we also found that the proportion of MDR-TB was significantly higher in the cavitary TB group than in the non-cavitary group (OR (95% CI): 2.53 (1.60–3.99), *P*<0.001).

In the multivariate analysis, the odds of cavitary vs non-cavitary TB were approximately 12 times greater (adjusted OR (95% CI), 12.08 (5.75–25.35), *P*<0.001) for patients with diabetes than for patients without diabetes and approximately 2.5 times higher (adjusted OR (95% CI), 2.48 (1.52–4.07), *P*<0.001) for MDR-TB patients than for non-MDR-TB patients (Table 2).

### Genotyping

Of the 385 MTB strains, 330 strains (85.7%) were classified as the Beijing genotype, whereas the other 55 (14.3%) belonged to the non-Beijing genotype. All of the Beijing genotype strains were further analyzed for the IS6110 insertion in the NTF region. Among the 330 Beijing genotype isolates, 260 (78.8%) belonged to the modern Beijing sublineage, and 70 (21.2%) belonged to the ancient Beijing sublineage.

**Figure 1:**
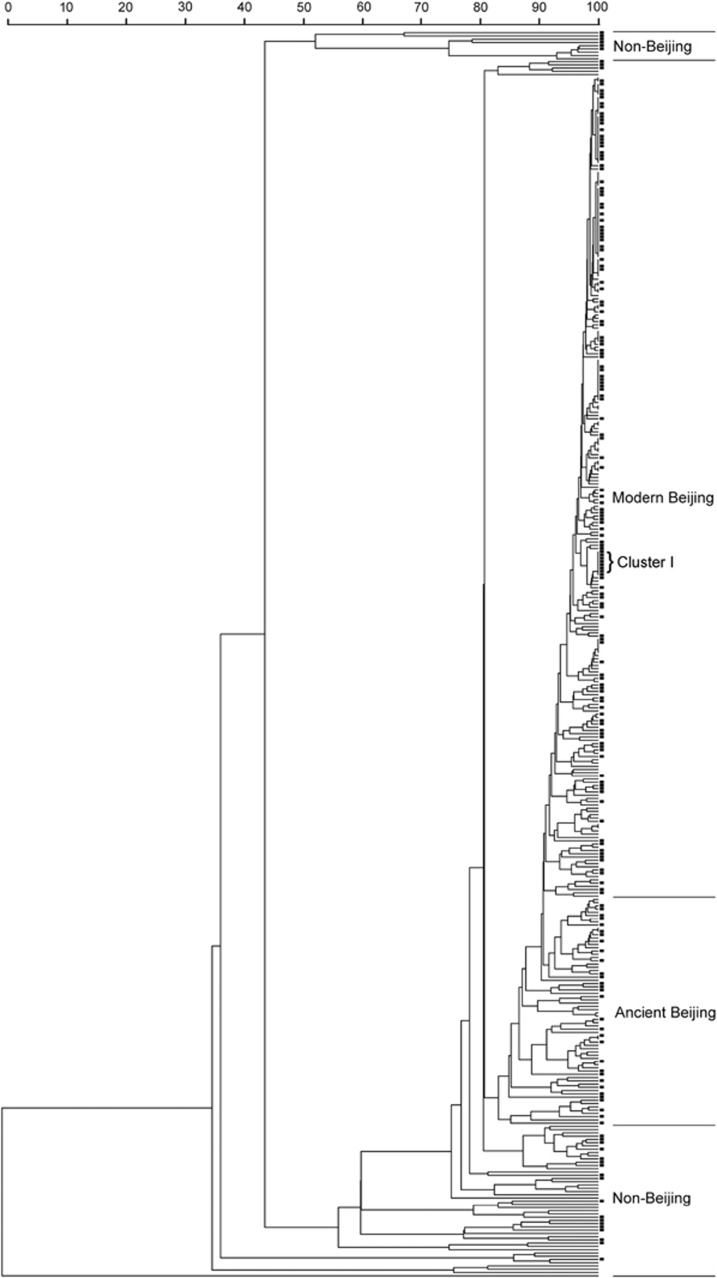
Dendrogram of the 385 MTB isolates. The phylogenetic tree was generated from the MIRU-VNTR profiles. The black square represents the isolates from the cavitary patients. Abbreviations: mycobacterial interspersed repetitive unit variable number of tandem repeats, MIRU-VNTR; *Mycobacterium tuberculosis*, MTB.

In addition, the 385 MTB strains were classified into 295 genotypes using the 15-locus VNTR method (Figure 1 and Supplementary Table S1). Among the 295 genotypes, 274 isolates harbored a unique pattern, whereas the other 111 isolates belonged to 21 clusters (2–22 isolates per cluster). The cumulative clustering rate was 23.4%, and the HGDI was 0.993 (Figure 1 and Table 3). We further analyzed the clustering rate among the different groups. As shown in Table 3, the clustering rate for cavitary TB (24.5%) was significant higher than that for non-cavitary TB (14.5%, *P*=0.019). Additionally, the clustering rates of the Beijing genotype and the non-Beijing genotype strains were 27.3% and 0.0%, respectively, and this difference was statistically significant (*P*<0.001). Similarly, the clustering rate of the modern Beijing strains was significantly higher than that of the ancient Beijing strains (33.9% vs 2.9%, *P*<0.001), which indicated that the higher clustering rate of the Beijing strains might be attributed to the high proportion of modern Beijing genotype strains in the current study.

The allelic diversity of each VNTR locus is shown in Table 4. The HGDI of Qub11b and Qub26 exceeded 0.6, which is classified as highly discriminating. Overall, the discriminating ability of the VNTR locus among the cavitary TB group was lower than the non-cavitary TB group, except for the MIRU40 and the exact tandem repeat A. Diversity was not observed for the ETRC to distinguish the cavitary and non-cavitary TB strains.

### Characteristics of the strains classified to the different genotypes

We further investigated the distribution of cavitary TB, the clustered strains and the MDR strains among the different genotypes. A comparison of the Beijing genotype strains from the cavitary and non-cavitary patients showed that 88.0% (169/192) of the strains in the cavitary patient group were of the Beijing genotype, which was not significantly different from the non-cavitary patient group (*P*=0.197; Table 5). In addition, 32.1% (106/330) of the Beijing genotype strains were MDR, which was significantly higher than the non-Beijing genotype strains (14.5%, *P*=0.008).

All of the clusters could be divided into large and small clusters according to the strain number of each cluster. As shown in Figure 1, there were 80 and 31 strains belonging to large and small clusters, respectively. Of the 80 strains from the large clusters, 51 (63.8%) were isolated from cavitary TB patients. Statistical analysis revealed that cavitary disease was observed more frequently among large clusters than among small clusters (*P*=0.037). Notably, all eight strains belonging to Cluster I caused cavitary diseases among the TB patients (Figure 1).

## Discussion

Pulmonary cavitation is the hallmark of TB disease and is responsible for delayed sputum culture conversion, poor clinical outcomes and infection transmission.^24^ In this study, our findings show that diabetes is significantly associated with the occurrence of cavitary TB. Previous studies have suggested that diabetes is a risk factor for MTB infection and TB development.^25^ In accordance with our study, a recent study by Chiang *et al.*^26^ revealed that diabetes influences the radiographic manifestations of pulmonary TB and that pulmonary TB patients with diabetes are more likely to present with cavitary TB than TB patients without diabetes.^25, 26^ Immune dysfunction in diabetes patients may result in the generation of pulmonary cavitation. Conversely, the higher bacillary burden at the cavity surface accelerates the expansion of the cavitation. As a result of rapid changes in lifestyle in China, the prevalence of diabetes has increased significantly in recent decades, with an estimated prevalence of 11.6% in the Chinese adult population in 2010.^27^ Considering the epidemic of both TB and diabetes in China, there is an urgent need for bidirectional screening for the two diseases to improve the diagnosis and management of dually affected patients, particularly to reduce the risk of developing cavitary TB.

In addition to diabetes, the multidrug resistance of the MTB strains was another important factor associated with cavitary disease. In MDR cases, the tubercle bacilli are resistant to anti-TB drugs, which results in the chronic progressive disease with cavitation in the lungs of patients.^14^ A previous study showed that the presence of pulmonary cavitation is linked to the development of drug resistance during treatment.^28^ Therefore, the interaction between pulmonary cavitation and drug resistance may accelerate the generation and expansion of pulmonary cavitation.

A previous meta-analysis described that younger TB patients are more likely to have cavities than elderly patients.^29^ In contrast to previous findings, our data demonstrated that patients aged 45–64 years had a higher risk of cavitary TB in the univariate analysis. The prevalence of diabetes is higher in older age groups,^27^ and the high prevalence of diabetes among the 45–64-year-old age group may be responsible for these conflicting results. In line with our hypothesis, a further multivariate analysis revealed that age was not an independent risk factor, which suggests that the potential confounding effect of age on diabetes influenced the univariate analysis results.

The Beijing genotype is the predominant MTB lineage in China, accounting for >60% of isolates.^4^ The successful spread of Beijing genotype has been attributed to its characteristics, including escaping from Bacille Calmette–Guérin vaccination and its association with drug resistance.^30, 31, 32^ In addition, several animal and macrophage models of pulmonary infection have demonstrated that the Beijing genotype has a higher virulence level *in vitro*.^32^ In this study, we found that there were no significant differences in the cavitary presentation between patients infected with the Beijing genotype compared with the non-Beijing genotype strains. Our results are in agreement with studies from Indonesia and The Netherlands,^33, 34^ in which the authors also did not find any significant difference between the groups. In contrast, a study performed in Singapore revealed that there was a significantly higher frequency of cavitary disease in patients infected with the non-Beijing genotype strains.^35^ It is well known that the Beijing genotype strains can be divided into several sublineages using other genotyping methods and that the different sublineages of the Beijing genotype strains show genotypic and phenotypic differences.^36^ One possible explanation for these contradictory conclusions may be that the prevalent Beijing genotype strains in the various geographic regions belong to different sublineages, which have adapted to the local host populations.^37^ Interestingly, this hypothesis was confirmed using a subsequent VNTR genotyping analysis, which showed that eight strains belonging to a cluster from the Beijing genotype all caused cavitary disease among TB patients. These results indicate that the MTB VNTR genotypes may be associated with the occurrence of TB cavitation. Similar results were observed in other mycobacteria species, which revealed that particular mycobacterial genotypes were more closely associated with disease progression and phenotype.^16, 17^ Cavitation is one of the most important clinical symptoms and has a strong association with treatment failure and relapse.^7^ The formation of cavitation depends on both bacterial virulence and host influence.^17^ Several studies have demonstrated that strains from the same cluster exhibit similar biological characteristics, including fitness, the ability to spread and pathogenicity.^36^ Therefore, the high proportion of cavitary disease among a specific cluster may be due to its high virulence, whereas synergy between bacterial virulence and host influence may explain the various proportions of cavitary disease among the other clusters. Based on our findings and the findings from previous studies, we hypothesized that the MTB genotypes based on the VNTR method would serve as a useful strategy to predict disease progression. In addition, it is important to characterize the molecular mechanisms responsible for the high virulence levels in the Cluster I MTB strains.

There were several obvious limitations of this study. First, it was conducted only in a TB specialized hospital, which may have biased the discriminatory power of the statistical analysis. Second, the correlation between cavitation and HIV status, another important comorbidity associated with poor immunity, was not analyzed in this study. Third, the host immune status of the patients was not collected in this study, which is essential to better understand the role of host immunity in the generation of cavitary diseases. Nevertheless, this study provides new insights into the factors that affect the occurrence of cavitary disease among TB patients.

In conclusion, our findings demonstrate that diabetes and multidrug resistance are risk factors associated with cavitary disease. In addition, there is no significant difference in the cavitary presentation between patients infected with the Beijing genotype strains and those infected with the non-Beijing genotype strains. Further studies will allow us to identify the molecular factors responsible for the increased risk of cavitary disease.

## Figures and Tables

**Table 1:** Demographic and clinical characteristics of the tuberculosis patients enrolled in this study

**Characteristics**	**Diagnostic class**				
	**Cavitary tuberculosis (192),** ***N*** **(%)**	**Non-cavitary tuberculosis (193),** ***N*** **(%)**	**Odds ratios (95% CI)**	***P*****-value**	**Total (385),** ***N*** **(%)**
Gender					
Male	119 (62.0)	123 (63.7)	1.00	—	242 (61.6)
Female	73 (38.0)	70 (36.3)	1.08 (0.71–1.63)	0.722	143 (38.4)
					
Age group (years)					
<25	31 (16.1)	40 (20.7)	1.00	—	71 (18.4)
25–44	51 (26.6)	59 (30.6)	1.12 (0.61–2.03)	0.721	110 (28.6)
45–64	68 (40.6)	29 (15.0)	3.03 (1.60–5.74)	0.001	97 (25.2)
>64	42 (21.9)	65 (33.7)	0.83 (0.45–1.53)	0.558	107 (27.8)
					
Treatment history					
New case	152 (79.2)	146 (75.6)	1.22 (0.76–1.98)	0.465	298 (77.4)
Re-treated	40 (20.8)	47 (24.4)	1.00	—	87 (22.6)
					
Comorbidity					
No	44 (22.9)	77 (39.9)	1.00	—	121 (31.4)
Diabetes	77 (40.1)	11 (5.7)	12.25 (5.89–25.48)	<0.001	88 (22.9)
Liver disease	23 (12.0)	31 (16.1)	1.30 (0.68–2.50)	0.434	54 (14.0)
Others	48 (25.0)	74 (38.3)	1.14 (0.68–1.91)	0.632	122 (31.7)
					
MDR					
Yes	75 (39.1)	39 (20.2)	2.53 (1.60–3.99)	<0.001	114 (29.6)
No	117 (60.9)	154 (79.8)	1.00	—	271 (70.4)

Abbreviations: confidence interval, CI; multidrug resistant, MDR.

**Table 2:** Multivariate analysis of the characteristics associated with cavitary tuberculosis in this study

**Characteristics**	**Cavitary vs non-cavitary**	
	**Adjusted OR (95% CI)**	***P*****-value**
Comorbidity		<0.001
No	1.00	
Diabetes	12.08 (5.75–25.35)	
Liver disease	1.30 (0.67–2.53)	
Others	1.13 (0.66–1.91)	
		
MDR		
Yes	2.48 (1.52–4.07)	<0.001
No	1.00	

Abbreviations: confidence interval, CI; multidrug resistant, MDR; odds ratio, OR.

**Table 3:** Discriminatory index and clustering rate of the 15-locus MIRU-VNTR set applied to the different *Mycobacterium tuberculosis* strains

**Isolates**	**Total number of isolates**	**Number of clustered isolates**	**Number of isolates in each cluster**	**Clustering rate (%)**	**HGDI**
Cavitary TB	192	56	2–14	24.5%	0.988
Non-cavitary TB	193	42	2–8	14.5%	0.997
					
Beijing genotype	330	111	2–22	27.3%	0.990
Ancient Beijing genotype	70	4	2	2.9%	0.999
Modern Beijing genotype	260	107	2–22	33.9%	0.985
					
Non-Beijing genotype	55	0	0	0.0%	1.000
Total	385	111	2–22	23.4%	0.993

Abbreviations: Hunter–Gaston Discriminatory Index, HGDI; mycobacterial interspersed repetitive unit variable number of tandem repeats, MIRU-VNTR; tuberculosis, TB.

**Table 4:** Allelic diversity of the 15 MIRU-VNTR loci among the *Mycobacterium tuberculosis* strains (*n*=385)

	**Locus**	**HGDI (all strains)**	**HGDI (cavitary tuberculosis)**	**HGDI (non-cavitary tuberculosis)**
1	Qub11b	0.690	0.690	0.692
2	Qub26	0.680	0.656	0.702
3	Mtub21	0.506	0.467	0.544
4	MIRU26	0.488	0.482	0.496
5	MIRU31	0.372	0.361	0.383
6	Mtub04	0.359	0.280	0.432
7	MIRU40	0.330	0.368	0.293
8	MIRU10	0.328	0.271	0.383
9	Mtub39	0.292	0.320	0.305
10	ETRA	0.285	0.300	0.272
11	Qub4156	0.228	0.203	0.252
12	Mtub30	0.216	0.230	0.203
13	MIRU04	0.177	0.140	0.212
14	MIRU16	0.162	0.158	0.167
15	ETRC	0.056	0.051	0.061

Abbreviations: exact tandem repeat A, ETRA; Hunter–Gaston Discriminatory Index, HGDI; mycobacterial interspersed repetitive unit variable number of tandem repeats, MIRU-VNTR.

**Table 5:** Differences in the *M. tuberculosis* characteristics among the Beijing and non-Beijing genotypes

**Characteristics**	**Number of isolates with different characteristics (%)**						
	**Beijing (*****n*****=330)**				**Non-Beijing (*****n*****=55)**	***P*****-value**	**Total (*****n*****=385)**
	**Ancient (*****n*****=70)**	**Modern (*****n*****=260)**	***P*****-value**	**Total**			
Cavitary	31	138	0.226	169	23	0.197	192
Non-cavitary	39	122		161	32		193
Clustered	4	107	<0.001	111	0	<0.001	111
Non-clustered	66	153		219	55		274
MDR	22	84	0.889	106	8	0.008	114
Non-MDR	48	176		224	47		271

Abbreviation: multidrug resistant, MDR.
